# Intralesional and Infusional Updates for Metastatic Melanoma

**DOI:** 10.3390/cancers16111957

**Published:** 2024-05-22

**Authors:** Michelle M. Dugan, Adrienne B. Shannon, Danielle K. DePalo, Matthew C. Perez, Jonathan S. Zager

**Affiliations:** 1 Department of Cutaneous Oncology, Moffitt Cancer Center, Tampa, FL 33612, USA; michelle.dugan@moffitt.org (M.M.D.); adrienne.shannon@moffitt.org (A.B.S.); dkdepalo@gmail.com (D.K.D.); matthew.perez@moffitt.org (M.C.P.); 2 Department of General Surgery, University of Massachusetts Chan Medical School, Boston, MA 01655, USA; 3 Department of Oncologic Sciences, University of South Florida Morsani College of Medicine, Tampa, FL 33602, USA

**Keywords:** metastatic melanoma, intralesional, isolated limb infusion, talimogene laherparepvec, melphalan, uveal melanoma, in-transit metastasis, regional chemotherapy

## Abstract

**Simple Summary:**

Many novel therapeutic options have emerged in recent years for patients with locoregionally advanced and metastatic melanoma. For in-transit melanoma with metastatic tumor spread between the primary tumor site and the regional lymph node basin, treatment options include intralesional tumor injections and an isolated infusion of the affected extremity. Notably, for ocular melanoma with metastatic disease to the liver, recent FDA approval of percutaneous hepatic perfusion, a minimally invasive method of isolated infusion of the liver, has become an approved treatment option. This article aims to review the current updates and efficacy on intralesional and infusional therapies for locoregionally advanced and metastatic melanoma.

**Abstract:**

Locoregionally advanced and metastatic melanoma represent a challenging clinical problem, but in the era of immune checkpoint blockade and intralesional and infusional therapies, more options are available for use. Isolated limb infusion (ILI) was first introduced in the 1990s for the management of advanced melanoma, followed by the utilization of isolated extremity perfusion (ILP). Following this, intralesional oncolytic viruses, xanthene dyes, and cytokines were introduced for the management of in-transit metastases as well as unresectable, advanced melanoma. In 2015, the Food and Drug Administration (FDA) approved the first oncolytic intralesional therapy, talimogene laherparepvec (T-VEC), for the treatment of advanced melanoma. Additionally, immune checkpoint inhibition has demonstrated efficacy in the management of advanced melanomas, and this improvement in outcomes has been extrapolated to aid in the management of in-transit metastatic disease. Finally, percutaneous hepatic perfusion (PHP), also approved by the FDA, has been reported to have a significant impact on the treatment of hepatic disease in uveal melanoma. While some of these treatments have less utility due to inferior outcomes as well as higher toxicity profiles, there are selective patient profiles for which these therapies carry a role. This review highlights intralesional and infusional therapies for the management of metastatic melanoma.

## 1. Introduction

Melanoma accounts for a large majority of skin cancer-related deaths and is one of the most common cancers in young adults [1]. The incidence of melanoma is rapidly increasing worldwide [2]. Melanoma can affect various anatomical sites, including the skin (cutaneous), mucosa (mucosal), or eye (uveal). While most patients with melanomas are diagnosed early, some patients are found to have metastatic disease at the time of diagnosis or develop metastatic disease at a later stage. 

The development of metastatic melanoma has significant prognostic implications. Five-year survival rates exceed 99% for localized disease, but can decrease to 74% for regional disease, and 35% for distant metastatic disease [1]. Up to 10% of patients with high-risk early-stage cutaneous melanoma will develop in-transit metastases (ITM), most often localized to the limbs, characterized by tumor nodules that spread along lymphatics in the subcutaneous or dermal layers between the primary lesion and regional nodal basin [3,4]. ITM without regional node involvement has been associated with 5-year survival rates of 60–69%, and ITM with regional node involvement has been associated with 5-year survival rates of 36–46% [3,5,6]. For patients with uveal melanoma who develop metastatic disease, approximately 90% will develop isolated liver metastases, with the liver being the predominant site of metastases in the majority of patients [4].

Internationally accepted treatment algorithms for treating extensive metastatic disease are currently lacking. When feasible, surgical excision represents a standard approach for isolated or localized disease with an overall low disease burden. Patients with more extensive disease may require other treatment modalities. The development of systemic immunotherapy and targeted therapy for metastatic melanoma has revolutionized disease management and prognosis, but systemic therapies can be associated with significant adverse events (AEs) [4,7]. Other novel approaches to metastatic disease include intralesional therapy and isolated limb infusion/perfusion therapy for ITM and percutaneous hepatic perfusion (PHP) for liver metastases. These therapies are usually used as monotherapy but have been reported to be used in combination with systemic therapy, especially in clinical trials. This review focuses on intralesional and infusion therapy options for cutaneous melanoma ITM, as well as hepatic perfusion therapy options for uveal melanoma liver metastases.

## 2. Intralesional Therapy for in-Transit Metastases

Intralesional therapies for advanced melanoma include injectable immune modulating therapies, gene therapies, peptide vaccines, and oncolytic viruses. These therapies can be either directly cytotoxic to tumor cells or can promote autologous tumor infiltration through an increased anti-tumoral immune response. Initial intralesional therapies included Bacille–Calmette–Guerin (BCG) and interferon-alpha (IFN-a). BCG, a live attenuated *Mycobacterium bovis* strain, when injected into tumors produces an inflammatory response and demonstrated an objective response in cutaneous metastatic melanoma [8]. However, this objective response failed to translate to improvements in overall survival (OS) and disease-free survival (DFS) when examined in a randomized controlled trial setting [9]. IFN-a has more traditionally been utilized as a systemic therapy in metastatic melanoma, but it has been utilized as an intralesional therapy with modest results [10]. While these historical options remain available, oncolytic viral therapy, such as talimogene laherparepvec, as well as melanoma vaccines, and immunomodulatory cytokines, appear to be more durable therapy options for ITM patients (Table 1).

### 2.1. Talimogene Laherparepvec

#### 2.1.1. Talimogene Laherparepvec as a Monotherapy

Talimogene laherparepvec (T-VEC) is an intralesional oncolytic herpes simplex virus type 1 (HSV-1) approved by the United States Food and Drug Association (FDA) in the treatment of advanced melanoma. In T-VEC, the HSV-1 has been genetically modified such that within the virus, the neurovirulence factors related to the ICP34.5 loci have been removed and coding for expression of granulocyte macrophage colony-stimulating factor (GM-CSF) has been included, resulting in selective replication within tumor cells, lysis, and local induction of a tumor-specific immune response [17]. T-VEC was approved following results from the international, phase 3 Oncovex^GM-CSF^ Pivotal Trial in Melanoma (OPTiM) trial, in which T-VEC demonstrated significantly improved overall survival (OS) in patients with stage IIIB-IVM1a melanoma treated with T-VEC compared to subcutaneous GM-CSF (median OS 23.3 vs. 18.9 months) [18]. Within OPTiM, 47% of injected lesions, 22% of non-injected non-visceral lesions, and 9% of non-injected visceral lesions demonstrated a complete response (CR) [18,19]. AEs included most commonly fatigue (50%) and an 11% incidence of grade 3/4 AEs, including cellulitis (2%) and vomiting (2%) [18]. The findings from OPTiM were mirrored in several single-institution and multi-institutional studies, with objective response rates (ORRs) of 57–79% and CR rates of 25–62% with similar AE profiles across anatomic sites [20,21,22,23,24,25]. Following these results, T-VEC has been recognized as the first and only approved agent in class for oncolytic viral therapy and has led to widespread use in the treatment of melanoma.

In 2021, a phase 2 randomized trial (NCT02211131) evaluating neoadjuvant T-VEC followed by surgery (n = 76) compared to surgery alone (n = 74) in resectable stage IIIB-IVM1a melanoma reported a 25% reduction in disease recurrence and a 51% reduction in disease-related mortality in the arm who received neoadjuvant T-VEC therapy compared to upfront surgery [13]. Additionally, the 2-year recurrence-free survival (RFS) as well as OS were improved for patients receiving neoadjuvant T-VEC (29.5% vs. 16.5%; 88.9% vs. 77.4%) compared to those patients who underwent upfront surgery [13]. The AE profile in this trial was similar to that of the previous OPTiM trial. 

While conventional immune checkpoint inhibitor (ICI) therapy can result in refractory disease, a recent single-institution study examining intralesional T-VEC reintroduction following disease recurrence noted the ongoing efficacy of the monotherapy and indicated T-VEC as a repeat therapy option for patients [26]. In this study, five patients with ITM who previously achieved histologically proven CR after a median of eight T-VEC courses developed recurrent ITM of the lower extremity after a time period between 3.8 and 14.2 months. All five patients achieved CR again after re-introduction of a median of five courses of T-VEC. Only one patient developed a second recurrence, and no patients developed distant metastases [26]. What is more, T-VEC remains a safe and effective therapeutic option for patients following failure of immunotherapy treatment, either sequentially or concurrently, with a reported ORR of 61% and a CR rate of 37% [27].

#### 2.1.2. Talimogene Laherparepvec in Combination with Other Treatment Modalities

Given that intralesional T-VEC induces a tumor-specific immune response, it has been hypothesized that simultaneous therapy with systemic ICI will produce a synergistic treatment effect [28]. An initial phase 1b trial evaluating T-VEC in combination with ipilimumab in patients with unresectable stage IIIB-IV melanoma reported an ORR of 50%, with 44% of patients having a durable response over 6 months [29]. A 2018 phase 2 trial (NCT01740297) noted that there was a 2.4-fold increase in CD8+ T cells in non-injected non-visceral lesions as well as increased expression of programmed death-ligand 1 (PD-L1) and cytotoxic T-lymphocyte-associated protein 4 (CTLA-4) [11,30]. A total of 98 patients with unresectable stage IIIB-IV melanoma treated with intralesional T-VEC plus ipilimumab, a monoclonal antibody that binds to CTLA-4, were compared to 100 patients who received ipilimumab alone; the ORR was significantly higher (39% vs. 18%) in the combination therapy group as compared to the monotherapy group [11]. On the 5-year follow-up study, median progression-free survival (PFS) was significantly improved following combination therapy (13.5 months vs. 6.4 months), and this response remained durable at the 5-year follow-up [30]. 

A phase 1b study evaluating intralesional T-VEC with pembrolizumab (Keytruda, Merck & Co., Inc., Rahway, NJ, USA), an anti-programmed cell death protein 1 (PD-1) antibody, noted an ORR of 62% and a CR rate of 33% with good tolerability [31,32]. Following this, a phase 3, randomized, double-blinded trial (NCT02263508) evaluating intralesional T-VEC with pembrolizumab as compared to intralesional placebo with pembrolizumab in 692 unresectable stage IIIB-IVM1c melanoma patients reported no difference in OS or PFS between the two cohorts [15]. A phase 2 trial with ongoing accrual evaluating intralesional T-VEC with pembrolizumab in anti-PD-1-refractory locally recurrent or metastatic melanoma reports preliminary results suggesting no efficacy (0% ORR) among patients with primary resistance and minimal efficacy (ORR 7%) among patients with acquired resistance [14]. However, this same trial reported reasonable efficacy among patients who progressed following receipt of anti-PD-1 therapy in the adjuvant setting (ORR 40–47%) [14]. The majority of AEs in this trial were pyrexia (30%) and fatigue (16%); there was a reported 13% of patients who developed grade 3 or higher AEs.

Numerous clinical trials examining the neoadjuvant use of T-VEC as a single agent and/or in combination with ICI are currently in progress. The phase 2 ongoing NIVEC trial (NCT04330430) is evaluating the rate of pathologic complete response in resectable stage IIIB-IVM1a melanoma following administration of neoadjuvant intralesional T-VEC and systemic nivolumab, an additional monoclonal antibody for PD-1, followed by surgical resection [33]. Additionally, neoadjuvant intranodal T-VEC injection combined with pembrolizumab is currently under evaluation in a phase 2 trial (NCT03842943) in patients with clinically node-positive melanoma. The addition of radiotherapy to T-VEC therapy is also being investigated. Currently, a phase 2 trial (NCT02819843) is underway comparing the outcomes of melanoma patients who receive intralesional T-VEC with or without the addition of radiotherapy. Lastly, a phase 1/2 trial (NCT03555032) is ongoing to evaluate the addition of isolated limb perfusion (ILP) to T-VEC therapy in stage IIIB-IVM1b melanoma. 

### 2.2. Antibody-Cytokine Fusion Proteins

#### 2.2.1. Interleukin-2

Interleukin-2 (IL-2), an endogenous immunomodulatory cytokine, is a well-tolerated injectable therapy that has demonstrated good objective response rates (ORRs) of 82% and complete response rates of 76–78% among patients with ITM [34,35]. However, IL-2 is not well tolerated systemically, resulting in the creation of Darleukin (L19IL2), a fusion protein of IL-2 to L19, a monoclonal antibody that targets an angiogenesis marker, allowing for preferential delivery of the IL-2 cytokine to tumor cells [36]. A phase 2 trial (NCT01253096) in 24 patients with unresectable stage IIIB/C melanoma treated with single-agent Darleukin demonstrated a CR rate of 25% with a more tolerable AE profile in which the most common toxicity was injection site pain (76%) [37]. Investigators are examining the role of the addition of intralesional Darleukin to systemic dacarbazine as compared to dacarbazine alone in stage IVM1a-b melanoma in a phase 1/2 trial (NCT02076646), which remains ongoing at this time.

#### 2.2.2. Daromun (L19IL2 and L19TNF)

Daromun is a combination of Darleukin and the fusion protein L19 and human recombinant tumor necrosis factor-alpha (L19TNF-α). The addition of L19TNF results in targeting of fibronectin expression and behaves synergistically to enhance antiangiogenic and antitumoral effects [38]. A phase 2 trial of weekly intralesional Daromun for 4 weeks in stage III-IVM1a melanoma noted, among 20 patients, an ORR of 55% and a DCR of 80% [39]. Similar to prior studies, the primary AE was injection site pain (73%); there were no grade 4/5 AEs reported. A current phase 3 trial (NeoDream, NCT03567889) is ongoing to evaluate Daromun as a neoadjuvant therapy in the management of stage IIIB/C melanoma [40]. NeoDream serves to determine the efficacy of intralesional neoadjuvant Daromun with systemic investigator’s choice ICI as compared to neoadjuvant systemic ICI alone. Similarly, a clinical trial (NCT02938299) is ongoing in Europe that is comparing intralesional neoadjuvant Daromun as compared to surgery alone. Some results are expected to be released at ASCO 2024.

### 2.3. PV-10

#### 2.3.1. PV-10 as a Monotherapy 

PV-10 is a solution of 10% Rose Bengal (RB,4,5,6,7-tetracholoro-2,4,5,7-tetraiodofluorescein disodium) xanthene dye that has been used for intralesional chemoablation of ITM. PV-10 results in tumor lysis, driving a local and systemic anti-tumoral immune response [41,42]. Additionally, PV-10 upregulates co-stimulatory markers on dendritic cells within the tumor; this maturation is associated with a response of non-injected lesions as well [43].

A phase 2 randomized clinical trial conducted with 80 patients with treatment-refractory American Joint Committee of Cancer 8th Edition stage III-IV melanoma noted an ORR of 51% with a CR rate of 26% [44]. Among non-injected lesions, the ORR was 33% [44]. AEs were primarily grade 1/2 and most commonly included injection site pain (80%) and edema (41%); there were no grade 4/5 AEs [44]. Similarly, three single-institution studies mirrored these findings with reported ORRs of 53–87% and CR rates of 26–46% with similar AE profiles [45,46,47]. These results in treatment-refractory patients, combined with the low-maintenance administration schedule of intralesional injections on day 0 and weeks 8, 12, and 16, represent an attractive option for well-selected patients. 

#### 2.3.2. PV-10 in Combination with Other Treatment Modalities

In 2017, Foote et al. conducted a phase 2, single-arm trial examining the addition of external beam radiotherapy (EBRT) to intralesional PV-10 in stage IIIB-IV melanoma at a single institution [48]. Fifteen patients were included in the trial with a total of 98 lesions; the study reported an ORR of 86.6% and a CR rate of 33.3% without grade 4/5 AEs [48]. Similar to prior studies, the most common AEs were grades 1 and 2 and included injection site pain (87%) and edema (60%) [48]. Their findings noted that a lesion size <1 cm was predictive of CR, suggesting a role for selection and perhaps earlier administration in the disease course [48]. 

Given that PV-10 upregulates co-stimulatory markers on dendritic cells within the tumor, resulting in tumor-specific T-cell activation, it has been hypothesized that the addition of systemic ICI may result in synergism and an improved therapeutic response. In mouse models, the addition of PD-1 inhibition to intralesional PV-10 therapy resulted in a reduction in the tumor burden and an increase in tumor-infiltrating lymphocytes (TIL), resulting in improved response rates [49]. 

At present, there is an ongoing phase 1b/2 trial (NCT02557321) investigating the addition of pembrolizumab to intralesional PV-10 in stage IIIB-IVM1c unresectable melanoma patients. Within this study, intralesional PV-10 is administered every 3 weeks with pembrolizumab for a maximum of 5 weeks, followed by continued pembrolizumab in isolation. The results from phase 1b included 21 ICI-naïve patients and reported an ORR of 67% with an AE profile of primarily grade 1/2 AEs with injection-related AEs associated with intralesional PV-10 as well as AEs traditionally attributed to ICI therapy [50]. A phase 1b expansion cohort performed among 14 ICI-refractory patients reported an ORR of 29% [51]. The phase 2 component of this trial, in which PV-10 with pembrolizumab compared to pembrolizumab alone remains ongoing. 

### 2.4. Tavokinogene Telseplasmid with Electroporation

Interleukin-12 (IL-12), a pro-inflammatory cytokine expressed by dendritic cells, macrophages, and neutrophils, stimulates interferon-gamma, regulates natural killer cells, and promotes T-cell maturation to drive T helper type 1 (Th-1) cell differentiation, resulting in a shift to cell-mediated immunity [52,53]. Systemic IL-12 is poorly tolerated and non-specific in the treatment of target melanoma lesions, but tavokinogene telseplasmid (tavo) is a plasmid that encodes IL-12 and allows for intralesional delivery [54]. A combination of this therapy with electroporation (EP) results in improved plasmid cell uptake and results in a transition in the tumor microenvironment from T helper type 2 (Th-2) to Th-1, driving innate and adaptive immunity [12,54]. Tumor regression following intralesional tavo-EP as well as immune infiltration has been noted in treated but also in untreated lesions, supporting evidence suggesting a shift in the tumor microenvironment in favor of adaptive immune resistance [12]. 

Intralesional tavo-EP was investigated in a phase 2 trial (NCT01502293) among 28 stage III-IV melanoma patients, with reports of an ORR of 36% and a CR rate of 18% [55]. The most common AEs were injection site reactions. Given that tavo-EP appears to drive a systemic adaptive immune response, it is hypothesized that systemic ICI should augment its efficacy on tumor lesions. As such, there is an ongoing phase 2 trial (NCT03132675) in 54 ICI-refractory patients with stage III/IV melanoma. Interim analysis of Keynote-695 reports an ORR of 30%, with tumor reduction uniformly noted in non-injected lesions as well [56]. The AE profile from this interim report notes overall mild AEs, primarily consisting of fatigue and injection site pain. Additionally, an ongoing phase 2 single-arm trial (NeoAd, NCT04526730) is investigating the efficacy of neoadjuvant intralesional tavo-EP in combination with nivolumab in resectable locally and regionally advanced melanoma patients. Patients received tavo-EP on days 1, 8, and 15 concurrently with 480 mg nivolumab on day 8 for up to three cycles, followed by surgical resection and adjuvant nivolumab. Interim results presented by Tarhini et al. in 2023 reported a preoperative ORR of 60% with a CR rate of 20% [16]. Among patients who underwent surgical resection, the major pathologic response rate was 78.6%; at median follow-up, no patient experienced disease recurrence, demonstrating promising efficacy in the management of these patients [16].

## 3. Isolated Limb Perfusion Therapy for in-Transit Metastases

**Figure 1:**
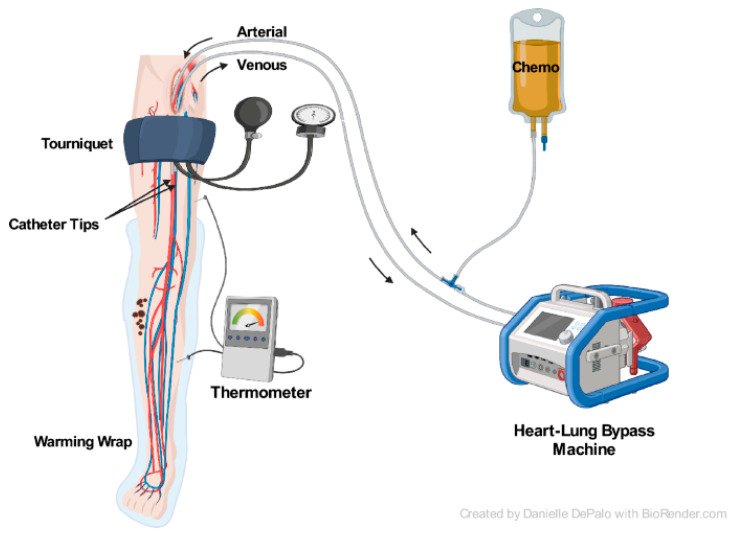
Isolated limb perfusion [4]. http://creativecommons.org/licenses/by/4.0/ (accessed on 1 April 2024) (no changes made).

ILP, first described by Creech and Krementz in 1958, involves surgical isolation of the affected extremity with extracorporeal circulation to deliver heated chemotherapy at high concentrations that would not be tolerated systemically (Figure 1) [57,58,59]. Continuous leakage monitoring and flow rate adjustments are conducted throughout the procedure to minimize the potential side effects of systemic escape from the chemotherapy [58]. The most common chemotherapy agent used for this perfusion is melphalan. In the setting of bulky or recurrent disease, the addition of tumor necrosis-factor alpha (TNF-alpha) can be valuable, although this agent is not available in North America or Australia [58]. 

ILP is a safe procedure with a low incidence of severe regional or systemic toxicity [60]. ILP has also been shown to be an effective treatment with excellent response rates. A large systematic review, including 22 studies with over 2000 ILPs, found a CR rate of approximately 60% and an ORR of approximately 90% [60]. Response rates seem to be independent of BRAF mutational status [61]. ILP can be safely repeated, such as in patients with recurrent disease who achieved CR after their first ILP or patients not responding to other therapies, achieving similar response rates as first-time ILP procedures [58,61]. Studies comparing response rates to ILP based on the prior receipt of systemic immunotherapy have shown conflicting results [62,63]. There are no studies showing that ILP decreases the risk of distant metastasis, and therefore ILP should be used as a locoregional treatment at this time [64,65].

## 4. Isolated Limb Infusion Therapy for in-Transit Metastases

**Figure 2:**
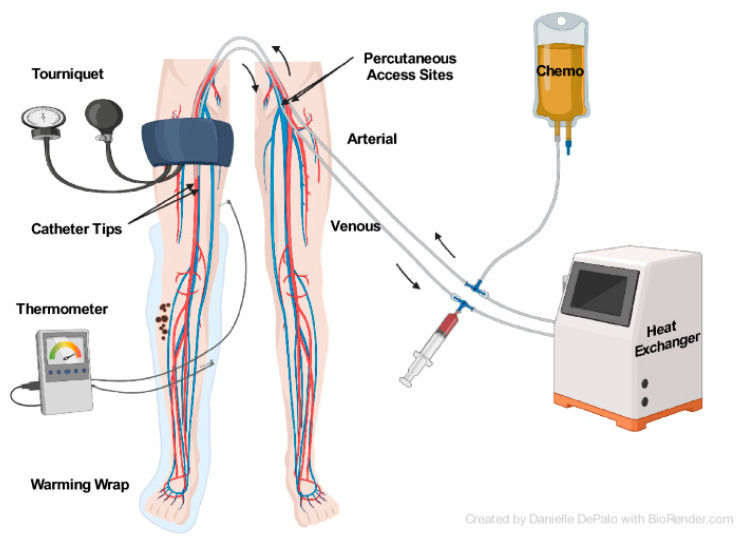
Isolated limb infusion [4]. http://creativecommons.org/licenses/by/4.0/ (accessed on 1 April 2024) (no changes made).

Isolated limb infusion (ILI), a less invasive adaptation of ILP, was described by Thompson et al. in 1996 [66]. Vascular access is gained through the percutaneous placement of arterial and venous catheters in the contralateral groin (Figure 2). Inflow and outflow occlusion are achieved with pneumatic tourniquet insufflation, in a similar fashion to ILP. A combination of heated melphalan and actinomycin D is typically used for the chemotherapy infusion. Melphalan is dosed according to limb volume and ideal body weight. Circulation continues for 30 min manually via a syringe [4,67]. 

ILI has shown excellent safety and efficacy. A large international multi-center study found a CR of 29% and an ORR of 64%, with a median overall survival greater than 6 years for complete responders [68]. ILI may be particularly useful in elderly patients, with one study finding an ORR of 70% in patients older than 80 years [69].

There have been no randomized comparisons between ILI and ILP reported, but some differences in outcomes and complication rates may be present (Table 2) [70,71,72,73,74]. ILP generally has higher ORRs than ILI reported in the literature [65,70,72,74]. In one of the most recent and largest studies, which adjusted for known predictive factors, a higher ORR for ILP (80% vs. 53%) and a higher CR for ILP (60% vs. 29%) were found, but there were no differences in overall survival outcomes [71]. Additionally, this study found no major differences in toxicity between each treatment modality [71]. Overall, both techniques require specialized resources and a multi-disciplinary team, and the recommended treatment modality will likely reflect what the particular institution is experienced with performing.

### Combination Therapies with ILP or ILI

It has been noted that ITM lesions typically have continued regression after ILP or ILI treatment and can take several months before metastases disappear completely. Therefore, it has been suggested that tumor regression is not solely due to the direct cytotoxic chemotherapy effects, but it also has an immune-mediated component. To further evaluate and potentially harness these synergistic effects, studies are combining ILI or ILP therapy with systemic immunotherapy. A phase 2 trial by Ariyan et al. combined ILI and ipilimumab in 26 patients and found an ORR of 85%, a CR of 62%, and a 1-year PFS of 58% [75]. This study suggested, based on murine models, that the synergistic effect was related to the heightened inflammation in the tumor microenvironment secondary to the local chemotherapy [75]. Ongoing clinical trials are currently evaluating the combination of ILP and nivolumab (Nivo-ILP trial, ClinicalTrials.gov: NCT03685890) (accessed on 1 April 2024) and the combination of ILP and T-VEC (TITAN trial, ClinicalTrials.gov: NCT03555032) (accessed on 1 April 2024).

## 5. Isolated Hepatic Perfusion for Uveal Melanoma Hepatic Metastases 

Uveal melanoma behaves very differently than cutaneous melanoma in terms of pathogenesis, incidence, clinical presentation, and response to treatment [4]. Distant metastatic disease is quite common even after control of the primary lesion, with distant metastasis rates of 25–31% within 5 years, 34–45% within 15 years, and 49% within 25 years of diagnosis [2,76]. For unclear reasons, over 90% of distant metastatic disease is within the liver, although dissemination to the lungs (24%), bone (16%), and soft tissues (11%), can also occur; however, this usually occurs later in the course of the disease, after liver involvement [77]. The prognosis is very poor for patients with uveal melanoma and distant metastatic disease, with a median OS of less than one year and a 2-year survival rate of 8% [78]. Although the overall tumor mutational burden is lower in uveal melanoma than cutaneous melanoma, a strong association has been found between distant metastatic disease and the BRCA-1 associated protein (BAP-1) mutation [79]. Rarely, patients with isolated liver metastases are candidates for surgical resection with negative margins [80]. Therefore, increasing emphasis has been placed on regional therapy options.

Isolated hepatic perfusion (IHP), a complex surgical technique first developed in 1960 by Ausman et al., is based on the principle of delivering highly concentrated regional chemotherapy directly to the isolated liver with minimal systemic toxicity [81]. Using an open surgical approach, complete vascular inflow and outflow are obtained, and the liver is perfused using a closed-circuit extracorporeal circuit [82,83]. The chemotherapeutic agent melphalan is typically used [83]. A major disadvantage of IHP is the morbidity associated with a complex open vascular/hepatobiliary operation. IHP has been used for liver tumors secondary to ocular melanoma, colorectal cancer, neuroendocrine tumors, cholangiocarcinoma, and hepatocellular carcinoma [84]. 

A long-term follow up study of 68 patients who received IHP for metastatic uveal melanoma found an ORR of 67%, a CR rate of 20%, and a median OS of 22.4 months [85]. Another study including 34 patients found an ORR of 68%, a CR rate of 12%, and a median OS of 24 months [86]. This study found a survival advantage of 14 months (*p* = 0.029) when comparing IHP to a control group of the longest surviving patients (based on registry data) not treated with IHP [86].

Although robust prospective literature on IHP efficacy has historically been lacking, a recent randomized control trial found superior outcomes in patients treated with IHP compared to best alternative care (BAC). The SCANDIUM trial, a multi-center randomized controlled phase 3 trial by Olofsson Bagge et al., investigated IHP in 93 patients with metastatic uveal melanoma [87]. Patients were randomly assigned to either IHP (n = 43) or a control group (n = 44) receiving the investigator’s choice of BAC treatment (49% chemotherapy, 39% immune checkpoint inhibitors, and 9% locoregional treatment other than IHP). The ORR was 40% for IHP versus 4.5% for the control group (*p* < 0.0001); the median PFS was 7.4 months for IHP vs. 3.3 months for controls (*p* < *0*.0001; HR 0.21 (95% CI: 0.12, 0.36); and the median hepatic PFS for IHP was 9.1 months vs. 3.3 months for controls (*p* < 0.0001) [87].

## 6. Percutaneous Hepatic Perfusion for Uveal Melanoma Hepatic Metastases

Percutaneous hepatic perfusion (PHP), developed in the 1990s, adapted the same principle from IHP of delivering high-dose chemotherapy to an isolated liver, but in a minimally invasive percutaneous fashion rather than an open surgical approach. Hepatic isolation is obtained using percutaneous catheters, with a double-balloon catheter in the retro-hepatic inferior vena cava via the femoral vein, a systemic venous return sheath in the internal jugular vein, and an infusion catheter in the hepatic artery via the femoral artery [80,88]. The venous outflow from the liver is run through an extracorporeal venous bypass circuit, filtering out any of the drug not absorbed by the liver, minimizing systemic exposure [88,89,90]. A phase 1 dose-escalation study using melphalan in patients with unresectable hepatic metastases found that 3 mg/kg is the maximum safe tolerated dose with the PHP technique and also found an ORR of 50% for patients with uveal melanoma [91]. The most common adverse events include anemia and thrombocytopenia secondary to hemofiltration, and bone marrow suppression [80,92,93].

PHP has been associated with significantly improved disease control compared to best alternative choice (BAC) therapy and excellent response rates. A multicenter randomized controlled trial by Hughes et al. analyzed 93 patients treated with either PHP or BAC and found a significantly better hepatic PFS (7 months vs. 1.6 months, *p* < 0.0001), overall PFS (5.4 months vs. 1.6 months, *p* < 0.0001), and a hepatic objective response (36.4% vs. 2%, *p* < 0.001) with PHP [93]. Another study including 51 patients who underwent PHP found a hepatic partial response (PR) of 43.1% and hepatic CR of 5.9%, with a hepatic disease control rate of 82.4%; the median overall PFS was 8.1 months; the median hepatic PFS was 9.1 months; and the median OS was 15.3 months [94]. A smaller study on 16 patients treated with PHP found a PR of 60%, stable disease (SD) of 33%, and progressive disease (PD) of 7%, with a median OS of 27.4 months [95].

PHP was recently approved by the FDA after the results of the phase 3 FOCUS trial [96]. In the FOCUS trial, 144 patients with hepatic-dominated metastatic ocular melanoma were randomized 1:1 to receive either PHP or BAC (the investigator’s choice of TACE, pembrolizumab, ipilimumab, or dacarbazine). The patients in the PHP arm could receive up to six PHP treatments, with each treatment repeated every six to eight weeks. Imaging was performed every 12 weeks to assess responses. The ORR for PHP was 35.2% compared to the ORR for BAC of 12.5% (*p* = 0.015). The disease control rate (DCR) for PHP was 73.6%, compared to the DCR for BAC of 37.5% (*p* = 0.0002). The median PFS for patients who underwent PHP was 9 months versus 3.1 months for BAC (*p* = 0.0007). The median OS for patients who underwent PHP was 20.5 months versus 14.1 months for BAC. Among the patients who underwent PHP, 42.6% experienced serious AEs, most of which were transient and hematological [96]. Overall, the response and disease control rates were both significantly better with PHP than BAC, and these results translated to a significantly prolonged PFS.

In a recent meta-analysis comparing IHP and PHP, there were no differences in OS (17.1 months for IHP vs. 17.3 months for PHP), PFS (7.2 months for IHP and 9.6 months for PHP), or hepatic PFS (10 months for IHP and 9.5 months for PHP) [97]. However, there was a significantly higher complication rate (39.1% vs. 23.8%) and a higher 30-day mortality rate (5.5% vs. 1.8%) for IHP when compared to PHP [97]. Another benefit of the PHP approach is the feasibility of repeating the therapeutic procedure, which is generally not possible with the surgical IHP approach.

Lastly, while the BAC arm in the FOCUS trial was made up of mostly TACE patients and PHP was significantly better in terms of liver and overall PFS, OS, and DCR, the only other retrospectively compared paper discussing TACE, Y90, and PHP is by Abbott et al. In this work, the authors showed in a small series of patients (30 patients: 6 Y90, 10 PHP, 12 CE, 1 PHP then Y90, and 1 CE then PHP) that PHP resulted in improved HPFS for PHP versus Y90 (*p* = 0.004), PHP versus CE (*p* = 0.02). PFS was also significantly improved in favor of PHP (Y90 (54 days), CE (52 days), and PHP (245 days), *p* = 0.03. PHP treatment and lower tumor burden were significant predictors of prolonged PFS on multivariate analysis. The median OS from time of treatment was the longest, but not significant, for PHP at 608 days versus Y90 (295 days) and CE (265 days). PHP treatment versus Y90 and lower tumor burden resulted in improved OS on multivariate analysis (*p* = 0.03, 0.03, respectively).

## 7. Conclusions

The outcomes for patients with locoregionally advanced or metastatic melanoma have improved significantly in the past few decades with the advent of various intralesional and infusion therapies. In patients with lesions amenable to the injection of intralesional agents, particularly those lesions that are unresectable, T-VEC as well as PV-10 and tavo-EP remain safe and effective options. The overall toxicity profile with these agents is mild, and previous studies have shown outcomes that offset the minimal AEs that occur. What is more, advances in the understanding of the tumor microenvironment and the interplay between the local tumor-specific immune response and the systemic immune response have opened the door for a variety of possibilities for patients with advanced melanoma. With the multitude of ongoing studies examining outcomes associated with combination immune therapy, results from these investigations will continue to shape the landscape for the management of in-transit disease in melanoma. 

Yet, there are limitations to intralesional therapy in that the therapy must be properly administered, and appropriate patient selection is important. Combination therapy with immune checkpoint blockade, targeted therapies, as well as radiotherapy, is still under investigation, and with advancements in gene profiling, additional targeted therapies may become of increased importance in the future. Beyond these therapies, infusion therapies, such as ILI and ILP, continue to play a large role in the management of advanced regional disease. These therapies, though more invasive, have shown reasonable results and are another tool to be utilized in this difficult patient population. Lastly, the inclusion of PHP as a treatment modality for patients with hepatic metastases from uveal melanoma has shown promising results.

## Figures and Tables

**Table 1:** Phase 2 or later clinical trials of intralesional therapies for advanced melanoma from 2018 to present. Abbreviations: IT, intratumoral. T-VEC, talimogene laherparepvec. ORR, objective response rate. PFS, progression-free survival. Tavo, tavokinogene telseplasmid. EP, electroporation. CR, complete response. OS, overall survival. RFS, recurrence-free survival. PD-1, programmed cell death protein 1. PR, pathologic response. IC, investigator’s choice. * denotes ongoing investigation/accrual not met.

	Year	Type	Treatment	Population	Cohort Size	Outcomes
NCT01740297 [11]	2018	Phase 2	IT T-VEC with ipilimumab verse ipilimumab alone	Unresectable stage IIIB-IV melanoma	Intervention arm N = 98, control arm N = 100	ORR 39% vs. 18%; PFS 13.5 months vs. 6.4 months
NCT01502293 [12]	2020	Phase 2	IT tavo-EP	Stage III/IV melanoma	N = 28	ORR 35.7%; CR 17.9%; median PFS 3.7 months; median OS 29.7 months; 46% had regression of at least one non-injected lesion
NCT02211131 [13]	2021	Phase 2	Neoadjuvant IT T-VEC with surgery vs. surgery alone	Resectable stage IIIB-IVM1a melanoma	Intervention arm N = 76, control arm N = 74	2-year RFS 29.5% vs. 16.5%; 2-year OS 88.9% vs. 77.4%
NCT04068181 [14]	2022 *, ongoing	Phase 2	IT T-VEC with pembrolizumab	Stage III/IV melanoma with previous progression/recurrence on anti-PD-1 therapy	Primary resistance cohort N = 26, acquired resistance N = 15, early recurrence N = 15, delayed recurrence N = 15	ORR 0%, 6.7%, 40%, and 46.7%, respectively, on preliminary analysis
NCT02263508 [15]	2023	Phase 3	IT T-VEC with pembrolizumab versus IT placebo with pembrolizumab	Unresectable stage IIIB-IVM1c melanoma	Intervention arm N = 346, control arm N = 346	ORR 48.6% vs. 41.3%; no difference in OS or PFS
NCT04526730 [16]	2023 *, ongoing	Phase 2	Neoadjuvant IT tavo-EP with nivolumab	Resectable stage III/IV melanoma	N = 17	ORR 60%; CR 20%; major PR 78.6%
NCT04330430	Ongoing	Phase 2	Neoadjuvant IT T-VEC and nivolumab	Resectable stage IIIB-IVM1a melanoma		
NCT03842943	Ongoing	Phase 2	Neoadjuvant intranodal T-VEC with pembrolizumab	Clinically node-positive melanoma		
NCT02819843	Ongoing	Phase 2	IT T-VEC with or without radiotherapy	Melanoma, Merkel cell carcinoma, and other solit tumor skin metastases		
NCT03555032	Ongoing	Phase 2	ILP and IT T-VEC	Stage IIIB-IVM1b melanoma		
NCT02076646	Ongoing	Phase 2	IT T-VEC with dacarbazine versus dacarbazine alone	Stage IVM1a-b melanoma		
NCT02557321	Ongoing	Phase 2	IT PV-10 with pembrolizumab	Unresectable stage IIIB-IVM1c melanoma		
NCT02557321	Ongoing	Phase 2	IT PV-10 with pembrolizumab verse pembrolizumab alone	Unresectable stage III/IV melanoma		
NCT03567889	Ongoing	Phase 3	Neoadjuvant Daromun and surgery and adjuvant IC immune therapy compared to surgery and adjvuant IC immune therapy alone	Stage IIIB/C melanoma		
NCT02938299	Ongoing	Phase 3	Neoadjuvant Daromun with surgery versus surgery alone	Stage IIIB/C melanoma		
NCT03132675	Ongoing	Phase 2	Tavo-EP with pembrolizumab	Immune-refractory stage III/IV melanoma		

**Table 2:** Comparison of isolated limb perfusion and isolated limb infusion techniques [4,70,71,72,73,74]. http://creativecommons.org/licenses/by/4.0/ (accessed on 1 April 2024) (minimal changes made).

	Isolated Limb Perfusion (ILP)	Isolated Limb Infusion (ILI)
Technique	Open operative vascular exposure	Percutaneous vascular catheterization
	3-h operative duration	1.5-h operative duration
	60-min perfusion time	20–30-min infusion time
	Heart–lung machine needed	Heart–lung machine not needed
	Fluoroscopy not needed	Fluoroscopy needed
	General anesthesia	Regional anesthesia possible
	TNF-alpha	TNF-alpha not used
	Leakage monitoring recommended	No leakage monitoring
	Repeatable	Repeatable
Outcomes		
Wieberdink grade IV toxicity	3–4%	0–1%
Overall response rate	80–81%	43–53%
Complete response rate	55–57%	24–50%
Median overall survival	33–40 months	32–46 months
